# Cutaneous Polyarteritis Nodosa in Childhood: A Case Report and Review of the Literature

**DOI:** 10.1155/2010/687547

**Published:** 2010-11-24

**Authors:** Nina-Karen Bansal, Kristin Michelle Houghton

**Affiliations:** Division of Rheumatology, British Columbia Children's Hospital, K4-123 ACB, 4480 Oak Street, Vancouver, BC, Canada V6H 3V4

## Abstract

Polyarteritis nodosa is a rare vasculitis of childhood. Cutaneous PAN (cPAN) is limited to the skin, muscles, joints, and peripheral nerves. We describe a 7.5-year-old girl with cPAN presenting initially as massive cervical edema who later went on to develop subcutaneous nodules, livedo reticularis, myositis, arthritis, and mononeuritis multiplex. The use of corticosteroids resulted in initial clinical improvement, but symptom recurrence necessitated disease modifying antirheumatic drugs and biologic therapy. We review a further 119 reports of biopsy proven cPAN in the literature. A majority of patients (96.6%) had cutaneous involvement; musculoskeletal involvement was common and included both articular (58.0%) and muscular (42.9%) symptoms, and nervous system involvement was least common (18.5%). Corticosteroids were used in the majority of patients (85.7%), followed by use of disease modifying antirheumatic drugs (33.0%), nonsteroidal anti-inflammatory drugs (10.7%), and intravenous immunoglobulin (9.8%). Therapy of cPAN with biologics has only been reported in 2 patients, and we report the first patient treated with Rituximab. A diagnosis of cPAN should be considered in a child with fever, vasculitic rash, and musculoskeletal symptoms. Most children respond to corticosteroids and have a benign course, but some require disease modifying antirheumatic drugs and biologic therapies.

## 1. Introduction

Polyarteritis nodosa (PAN) is a rare vasculitis in childhood. Since first described by Kussmaul and Maier in 1866 [1], there have been approximately 200 pediatric case reports in the literature. Traditionally, children were classified as having one of three forms: infantile, cutaneous, and systemic. Infantile PAN is now recognized as a severe form of Kawasaki disease. Criteria for a diagnosis of systemic PAN in childhood have been proposed but not validated [2]. 

Cutaneous PAN (cPAN) is recognized as a separate entity but there are no diagnostic criteria for cPAN. [2] cPAN is characterized by disease affecting the skin with no major organ system involvement. The cutaneous symptoms are similar to systemic PAN and mild fever, muscle, joint, and peripheral nervous system involvement may also occur. Fever, rash, and musculoskeletal symptoms are common in children and cPAN needs to be differentiated from other diagnostic entities. Definitive diagnosis is by histopathologic evidence of necrotizing inflammation of the medium and small-sized arteries. There is a paucity of knowledge of the spectrum of clinical presentation and management of children with cPAN. We describe a severe case of cPAN and summarize the clinical manifestations, laboratory data and treatment regimens of our patient as well as those reported in the literature. To our knowledge, we report the first patient with childhood cPAN treated with Rituximab, and we report the largest pediatric review of cPAN.

## 2. Materials and Methods

This is a case study and review of the literature of childhood cPAN with analysis of the clinical and laboratory features and treatment practice. 

### 2.1. Literature Review

All case reports and case series of PAN in childhood reported in the English Literature between 1950 and 2009 identified through Medline were reviewed. There is no strict definition of cPAN and we included all reports of children aged 18 and younger with skin involvement, absence of visceral involvement, and biopsy evidence of necrotizing inflammation of small and medium-sized arteries.

Exclusion criteria were the presence of another identified vasculitis including systemic PAN, infantile PAN, microscopic polyangiitis, and Hepatitis B-associated PAN.

Data collection, by chart review or extraction of information from individual publications, included demographic, clinical, laboratory, and treatment information. Few reports have complete data; therefore, depending on the availability for each reported variable, the denominator used for analysis varies. The following clinical and laboratory measures were recorded: skin, muscle, joint and nervous system involvement, antistreptolysin O titre (ASOT), throat swab, and hepatitis B serology. Treatment was classified into one of the following groups: corticosteroid, nonsteroidal anti-inflammatory drug (NSAID), acetylsalicylic acid (ASA), disease modifying antirheumatic drug (DMARD), intravenous immunoglobulin (IVIG), biologic therapy, penicillin, or other medication.

### 2.2. Statistical Analysis

Descriptive statistics were calculated for demographic, clinical, laboratory, and treatment data. Medians, ranges, and percentages are presented as appropriate.

### 2.3. Case Report

A 7.5-year-old previously healthy female of mixed Caucasian-Middle Eastern descent presented to the emergency department with unilateral mandibular swelling and neck pain on passive movement. She had been prescribed erythromycin for 3 days of pharyngitis and 1 day of fever. She did not have hoarseness, drooling, or respiratory distress. There was no recent travel, TB exposure, or animal contact. Her father had recently been treated for a streptococcal pharyngitis; otherwise there were no ill contacts. 

Past medical history was unremarkable. She was fully immunized. 

On examination she looked well. She was febrile at 38.2°C; the rest of her vital signs were normal. She had soft tissue swelling along the left mandible extending down the neck. She had mild left cervical adenopathy that was tender to palpation. She was unable to fully flex and rotate her neck due to pain. The remainder of the examination was unremarkable. Complete blood count revealed leukocytosis (29.7 × 10^9^/L) with neutrophilia (26.34 × 10^9^/L), normal hemoglobin (118 g/L), and thrombocytosis (466 × 10^9^/L). CRP and ESR were elevated at 77 mg/L and 47 mm/hr, respectively. Electrolytes, renal function, and amylase were normal. Lateral neck X-ray revealed mild thickening of the prevertebral soft tissues. She was diagnosed with cervical adenitis and admitted for antibiotic therapy. 

After admission, she clinically deteriorated. She had daily fevers and the edema increased and spread to the right mandible and anterior chest wall. On day 5 she developed a pruritic papular erythematous rash over her chin, neck, chest, and back. She had palmar erythema and erythematous papules on the dorsum of both feet. On day 8 she developed arthralgias of the left knee and ankles, bilateral calf pain, localized bone pain over the diaphysis of her right tibia, and pain over the dorsum of both feet. She had bilateral ankle effusions and was unable to weight bear. 

Her clinical course was further complicated by acute renal failure, presumed to be secondary to combined vancomycin, and NSAID nephrotoxicity. She also had an episode of hematemesis, mild hepatitis, and coagulopathy.

**Figure 1:**
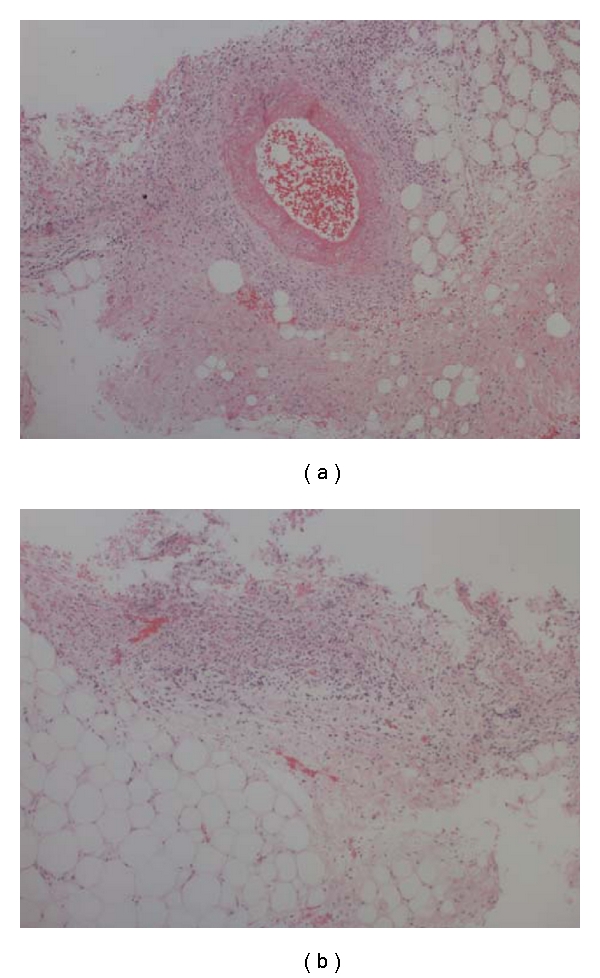
Histopathology of a vasculitic lesion from the anterior chest wall shows septal panniculitis with leukocytoclastic vasculitis involving a small artery consistent with cPAN. No granulomas or microorganisms were seen. (Figure courtesy of Oana-Eugenia Popescu, MD, Division of Anatomical Pathology, Children's and Women's Health Centre of BC).

ESR, CRP, and platelet count were persistently elevated, indicative of an extensive inflammatory process. Bacterial cultures of blood, throat (Group A Strep), and lymph node aspirate were negative. Serology for CMV, EBV, HHV-6, hepatitis A, B and C, and Toxoplasmosis were negative. Antistreptolysin O titre was elevated (643 IU/mL), suggesting a poststreptococcal phenomenon. IgG titre to *Bartonella henselae* was elevated at 1 : 320, of unclear significance. ANCA, ANA, ENA, and dsDNA were all negative. An echocardiogram showed normal cardiac anatomy and function. Ultrasound and CT of the neck and chest demonstrated moderate lymph node enlargement and soft tissue edema, but no abscess or lymphatic mass. Bronchoscopy revealed 50% pharyngeal narrowing and was complicated by acute upper airway compromise that improved with oral dexamethasone 0.3 mg/kg. Ultrasound of her lower extremities was negative for deep vein thrombosis but demonstrated left knee effusion. A skin biopsy of a vasculitic lesion from the anterior chest wall revealed septal panniculitis with leukocytoclastic vasculitis involving the small- and medium- sized blood vessels consistent with cPAN. No granulomas or microorganisms were seen (Figure 1). 

Her treatments included prolonged intravenous antibiotics with no effect (cefazolin days 1 to 3, cefuroxime and clindamycin days 3 to 5, vancomycin days 5 to 13, pipercillin-tazobactam days 5 to 18). IVIg (2 g/kg) was given (on day 11) for possible atypical Kawasaki disease without effect. Her hematemesis and coagulopathy responded to pantoprazole and vitamin K. After review of the skin biopsy results, she was treated with pulse doses of intravenous methylprednisolone (30 mg/kg/day) (days 13 to 15), then moderate dose oral prednisone 1 mg/kg/day. Her rash, edema, and arthritis quickly responded to corticosteroids. On day 21 she was discharged on oral prednisone. 

Her fever, rash, and leg pain recurred when prednisone was tapered. NSAIDs were initially helpful, but she soon required admission for intravenous analgesia. On examination she was febrile and hypertensive. She had polyarthritis. Her neurologic exam revealed spastic catch at both ankles with diminished strength (4 − 4 + /5) in hip flexion and extension, and knee extension. Gower's sign was positive. Cerebellar exam was normal but on gait testing she toe-walked and was unable to perform tandem gait. Skin examination revealed palmar and palatal erythema. She had livedo reticularis and subcutaneous nodules on her arms and legs.

Nerve conduction studies demonstrated diminished amplitude of the left peroneal and right median motors; diminished conduction velocity of the tibial motors bilaterally; and absent F response in the left peroneal motor. These findings were compatible with mononeuritis multiplex. MRI of the spine and renal angiogram were normal. She was treated with pulse doses of intravenous methylprednisolone (30 mg/kg), intravenous cyclophosphamide (500 mg/m^2^), IVIg (2 g/kg), naproxen, gabapentin, and amlodipine. Her symptoms improved, and she was discharged on day 8, on high dose oral prednisone, monthly cyclophosphamide infusions (1000 g/m^2^), naproxen and gabapentin.

She was maintained on monthly infusions of cyclophosphamide (1 g/m^2^) and methylprednisolone and was able to slowly wean gabapentin, naproxen, and oral prednisone. After 8 months, cyclophosphamide infusions were stretched to every 2 months. However, she had a flare of her disease that necessitated admission to hospital. After a 10th dose of cyclophosphamide she was started on subcutaneous methotrexate 0.5 mg/kg weekly and cyclophosphamide was discontinued to minimize cumulative cyclophosphamide exposure. After 1 month, because of persistent symptoms, methotrexate was increased to 0.75 mg/kg and monthly Infliximab (5 mg/kg) infusions were started. Infliximab was increased to 10 mg/kg/dose q 4 weeks, and she was maintained on moderate dose prednisone, methotrexate, and naproxen. She remained corticosteroid dependent and had a flare of her arthritis, rash, fever, and mononeuritis multiplex with weaning of prednisone below 0.5 mg/kg/day. After 8 doses of infliximab she remained corticosteroid-dependent and a decision was made to stop infliximab and start rituximab. Our patient did not have any positive autoantibodies and rituximab was chosen as it has been reported to be of benefit in recalcitrant pediatric vasculitides. [3] She received rituximab (375 mg/m^2^) and cyclophosphamide (500 mg/m^2^) at days 1 and 15. She has done well since receiving rituximab and has tolerated weaning of prednisone.

## 3. Results

Review of the literature identified 582 cases of PAN, including 140 cases of cPAN in case reports and small case series. 463 were excluded: systemic PAN (327), infantile PAN (37), hepatitis B-associated PAN (24), microscopic polyangiitis (53), other vasculitis (1), and cPAN with absence of histopathological confirmation of the diagnosis or incomplete clinical datasets (21). 119 cases of cPAN met our inclusion criteria of age 18 and younger with skin involvement, absence of visceral involvement, and biopsy evidence of necrotizing inflammation of small and medium sized arteries.

Clinical information for the 119 cases of cPAN is summarized in Table 1. The female : male ratio was 1.2 : 1. The median age was 10 years (range 7 months to 18 years). As expected, the majority of patients (96.6%) exhibited cutaneous symptoms, including subcutaneous nodules, livedo reticularis, purpura, edema, and/or ulcers. Musculoskeletal involvement was common with arthralgias and/or arthritis reported in 58.0% and myalgias and/or myositis in 42.9% of cases. Nervous system involvement was least common with 18.5% of cases reporting mononeuritis multiplex, peripheral neuropathy, cranial nerve palsy, and/or abnormal nerve conduction. All 119 patients had documented skin or muscle biopsies demonstrating necrotizing vasculitis of the small- and medium-sized arteries. 

Testing for group A streptococcal infection was positive in 86.2% of cases (50 of 58 tested), as manifested by elevated ASOT and/or positive throat swab. 

Therapy is summarized in Table 2. Corticosteroids were used in the majority of patients (85.7%). Various DMARDs, including azathioprine, cyclophosphamide, methotrexate, and mycophenolate mofetil were used in (33.0%) of patients. NSAIDs were prescribed in 10.7% of cases, ASA in 10.7%, and IVIG in 9.8%. Therapy of cPAN with biologics (infliximab, etanercept) has only been reported in 2 patients [3]. Other therapies were used in 10.7% of cases and included colchicine, dapsone, oxpentifylline, ACTH, and platonin.

## 4. Discussion

cPAN is not common in the pediatric population with approximately 140 cases reported in the literature. Disease is limited to skin, joints, and muscles in the majority with a minority having nerve involvement. Constitutional symptoms are common. Most children have a chronic and relapsing benign course.

We report a severe case of cPAN in a 7.5-year-old girl. Our patient eventually responded to rituximab after not responding to multiple corticosteroid sparing immunosuppressive therapies. She remains corticosteroid dependent after 24 months. Consistent with the published literature she is responsive to high-dose corticosteroids, but had disease relapse as corticosteroids were weaned. Importantly, she has not developed visceral involvement and has not evolved to systemic PAN. 

Cutaneous and systemic PAN share the same histopathologic features of necrotizing arteritis of small and medium sized vessels. Kussmaul and Meier described the first cases of systemic PAN in 1866 [1]. Early reports [6, 54] confirm that cPAN is a separate entity to systemic PAN. We have limited our definition of cPAN to disease affecting the skin, muscle, joints, and peripheral nervous system, with corresponding biopsy confirmation. Any evidence of visceral involvement, either clinically (central nervous system, pulmonary, cardiac, gastrointestinal, or renal), radiographically (abnormal angiography), or by histology (visceral biopsy) were classified as systemic PAN. Nakamura et al. [17] proposed further restriction of the definition of cutaneous PAN in that any extracutaneous involvement such as peripheral neuropathy and myalgias must be limited to the same area as skin lesions. Systemic PAN and cPAN appear to be fairly distinct entities on a clinical continuum. There are only 5 reported cases of cPAN evolving into systemic PAN [55, 56].

On review of treatment regimens reported in the literature, most children respond to corticosteroids. Penicillin should be considered in children with increased ASO titres [5, 7, 9, 16, 56]. Recent case series report success with low-dose methotrexate, cyclophosphamide, intravenous immunoglobulin, and biologic therapies [3, 4, 11, 57]. Eleftheriou and colleagues report the largest cohort of pediatric patients treated with biologic therapy. Of the 25 patients reported, 11 had PAN and 2 had cPAN as per our case definition of skin involvement and the absence of visceral involvement. Both patients with cPAN responded to anti-TNF therapy; one to infliximab and one to etanercept. Overall, 8 patients with systemic PAN received infliximab and three received etanercept. Two patients with systemic PAN required further treatment with a second biologic agent, one each with rituximab and adalimumab. Five other patients (unclassified vasculitis (*n* = 4) and Wegener granulomatosis (*n*  =  1)) also required treatment with a second biologic agent (rituximab (*n* = 2), infliximab, etanercept and anakinra (*n* = 1). Of concern, infectious complications occurred in 24% of patients. The authors concluded that biologic therapy may be helpful in treating primary systemic vasculitis and recommend children with PAN who fail standard therapy or have high cumulative cyclophosphamide dose be treated with rituximab or anti-TNF therapy [3].

Our patient had a raised ASO initially, but she did not receive ongoing Penicillin prophylaxis. She was initially treated with high-dose corticosteroids and cyclophosphamide as standard recognized therapy for severe vasculitis. She did well on cyclophosphamide but did not tolerate weaning from monthly to 2 monthly infusions and was switched to infliximab and methotrexate to minimize cumulative cyclophosphamide dose. Consistent with the literature, she initially responded to infliximab but was switched to rituximab with cyclophosphamide due to failure to control disease activity despite high dose (10 mg/kg) and frequent infliximab infusions (every 4 weeks). Of interest she did not have any positive autoantibodies, and the literature suggests efficacy of rituximab is not confined to antibody positive patients [3]. Currently she is maintained on 5mg prednisone and methotrexate. On her current therapy she is doing well and has returned to school and regular physical activity. 

There are inherent limitations of a retrospective study of cases reported in the literature over a 60 year time period. Many of the case series did not report individual characteristics; therefore the data could not be extracted on individual patients. Immunosuppressive therapies have changed over time and biologic therapies appear promising for children who fail standard therapies. 

In summary, cPAN can be challenging to diagnose and manage. A diagnosis of cPAN should be considered in a child with fever, tender subcutaneous nodules, livido reticularis, and arthralgias/arthritis. Most children respond to corticosteroids and have a benign course, but some children may be corticosteroid dependent or corticosteroid resistant, necessitating other immunosuppressive agents including DMARDs and biologic therapies. There is no firm choice of first-line biologic therapy for childhood PAN, and we report a case of cPAN responsive to rituximab after failing TNF inhibition. Multicentre pediatric vasculitis disease registries are necessary to inform development and standardization of best clinical practice for childhood cPAN.

## Figures and Tables

**Table 1:** Frequency of system involvement in childhood cPAN (*N* = 119).

Study	Total (*N*)	Skin involvement (Subcutaneous nodules, livido reticularis, purpura, edema, and/or ulcers)	Joint involvement (Arthralgia, arthritis)	Muscle involvement (Myalgias, myositis)	Nerve involvement (Mononeuritis multiplex, peripheral neuropathy, cranial nerve palsy, and/or abnormal nerve conduction)
Ozen et al. [4]	*N* = 33	33 (100%)	13 (39%)	5 (15%)	1 (3%)
Kumar et al. [5]	*N* = 9	9 (100%)	7 (78%)	0 (0%)	0 (0%)
Diaz-Perez et al. [6]	*N* = 5	5 (100%)	3 (60%)	5 (100%)	3 (60%)
Fink [7]	*N* = 5	5 (100%)	5 (100%)	5 (100%)	0 (0%)
Tinaztepe et al. [8]	*N* = 4	4 (100%)	4 (100%)	4 (100%)	0 (0%)
Sheth et al. [9]	*N* = 4	4 (100%)	4 (100%)	4 (100%)	2 (50%)
Topaloglu et al. [10]	*N* = 4	3 (75%)	2 (50%)	3 (75%)	4 (100%)
Fathalla et al. [11]	*N* = 4	4 (100%)	3 (75%)	0 (0%)	0 (0%)
Kirkali et al. [12]	*N* = 3	2 (67%)	0 (0%)	1 (33%)	3 (100%)
Yonis [13]	*N* = 3	3 (100%)	0 (0%)	3 (100%)	2 (67%)
Siberry et al. [14]	*N* = 2	2 (100%)	2 (100%)	0 (0%)	0 (0%)
Finkel et al. [15]	*N* = 2	2 (100%)	2 (100%)	2 (100%)	1 (50%)
Eleftheriou et al. [3]	*N* = 2	2 (100%)	0 (0%)	1 (50%)	1 (50%)
Till et al. [16]	*N* = 2	2 (100%)	2 (100%)	0 (0%)	0 (0%)
Single case reports [17–53]	*N* = 37	*N* = 35 (95%)	*N* = 22 (59%)	*N* = 18 (49%)	*N* = 5 (14%)

TOTAL	*N* = 119	115 (96.6)	69 (58.0)	51 (42.9)	22 (18.5%)

**Table 2:** Frequency of therapies used in management of childhood cPAN (*N* = 112).

Medication*	*N* (%)	References
Corticosteroid	96 (85.7)	[4, 5, 7–14, 16, 21, 22, 24–35, 39–41, 43–51, 53]
DMARD (azathioprine, cyclophosphamide, methotrexate, and mycophenolate mofetil)	37 (33)	[4, 5, 8, 10–12, 15, 21, 22, 29, 30, 34, 37, 45, 47–50, 53]
Penicillin	33 (29.5)	[7, 9, 11, 13, 14, 16, 24, 37, 41, 42, 44, 46, 47]
ASA	12 (10.7)	[9, 14, 24, 29, 31–33, 38, 42, 45]
NSAID	12 (10.7)	[4, 9, 11, 16, 24, 26, 28, 31, 32, 37, 41]
IVIG	11 (9.8)	[9, 11, 14, 15, 35, 36, 42, 45, 51, 53]
Biologic (infliximab, etanercept)	2 (1.8)	[3]
Other (colchicine, dapsone, oxpentifylline, ACTH, and platonin)	12 (10.7)	[8, 13, 18, 29, 53]

*Used at anytime throughout disease management

## References

[1] Kussmaul A, Maier R (1866). Über eine bisher nicht beschriebene Arterienerkrankung (Periarteritis nodosa), die mit Morbus Brightii und mit rapid fortschreitender allgemeiner Muskellähmung einhergeht. *Deutsches Archiv für Klinische Medizin*.

[2] Ozen S, Pistorio A, Iusan SM (2010). EULAR/PRINTO/PRES criteria for Henoch-Schönlein purpura, childhood polyarteritis nodosa, childhood Wegener granulomatosis and childhood Takayasu arteritis: Ankara 2008. Part II: final classification criteria. *Annals of the Rheumatic Diseases*.

[3] Eleftheriou D, Melo M, Marks SD (2009). Biologic therapy in primary systemic vasculitis of the young. *Rheumatology*.

[4] Ozen S, Anton J, Arisoy N (2004). Juvenile polyarteritis: results of a multicenter survey of 110 children. *Journal of Pediatrics*.

[5] Kumar L, Thapa BR, Sarkar B, Walia BNS (1995). Benign cutaneous polyarteritis nodosa in children below 10 years of age—a clinical experience. *Annals of the Rheumatic Diseases*.

[6] Diaz-Perez JL, Winkelmann RK (1974). Cutaneous periarteritis nodosa. *Archives of Dermatology*.

[7] Fink CW (1991). The role of the streptococcus in poststreptococcal reactive arthritis and childhood polyarteritis nodosa. *Journal of Rheumatology*.

[8] Tinaztepe K, Gucer S, Bakkaloglu A, Tinaztepe B (1997). Familial Mediterranean fever and polyarteritis nodosa: experience of five paediatric cases. A causal relationship or coincidence? [1]. *European Journal of Pediatrics*.

[9] Sheth AP, Olson JC, Esterly NB (1994). Cutaneous polyarteritis nodosa of childhood. *Journal of the American Academy of Dermatology*.

[10] Topaloglu R, Besbas N, Saatci U, Bakkaloglu A, Oner A (1992). Cranial nerve involvement in childhood polyarteritis nodosa. *Clinical Neurology and Neurosurgery*.

[11] Fathalla BM, Miller L, Brady S, Schaller JG (2005). Cutaneous polyarteritis nodosa in children. *Journal of the American Academy of Dermatology*.

[12] Kirkali P, Topaloglu R, Kansu T, Bakkaloglu A (1991). Third nerve palsy and internuclear ophthalmoplegia in periarteritis nodosa. *Journal of Pediatric Ophthalmology and Strabismus*.

[13] Yonis IZ (1959). Periarteritis nodosa; report of three cases successfully treated by cortisone and A. C. T. H. *Annales Paediatrici*.

[14] Siberry GK, Cohen BA, Johnson B (1994). Cutaneous polyarteritis nodosa: reports of two cases in children and review of the literature. *Archives of Dermatology*.

[15] Finkel TH, Török TJ, Ferguson PJ (1994). Chronic parvovirus B19 infection and systemic necrotising vasculitis: opportunistic infection or aetiological agent?. *The Lancet*.

[16] Till SH, Amos RS (1997). Long-term follow-up of juvenile-onset cutaneous polyarteritis nodosa associated with streptococcal infection. *British Journal of Rheumatology*.

[17] Nakamura T, Kanazawa N, Ikeda T (2009). Cutaneous polyarteritis nodosa: revisiting its definition and diagnostic criteria. *Archives of Dermatological Research*.

[18] Weller SD (1953). Periarteritis nodosa. *Proceedings of the Royal Society of Medicine*.

[19] Boren RJ, Everett MA (1965). Cutaneous vasculitis in mother and infant. *Archives of Dermatology*.

[20] Dyer NH, Verbov JL, Dawson AM, Borrie PF, Stansfeld AG (1970). Cutaneous polyartheritis nodosa associated with Crohn’s disease. *The Lancet*.

[21] Verbov J, Stansfeld AG (1972). Cutaneous polyarteritis nodosa and Crohn’s disease. *Transactions of the St. John"s Hospital Dermatological Society*.

[22] Reimold EW, Weinberg AG, Fink CW, Battles ND (1976). Polyarteritis in children. *American Journal of Diseases of Children*.

[23] Kahn EI, Daum F, Aiges HW, Silverberg M (1980). Cutaneous polyarteritis nodosa associated with Crohn’s disease. *Diseases of the Colon and Rectum*.

[24] Jones SK, Lane AT, Golitz LE, Weston WL (1985). Cutaneous periarteritis nodosa in a child. *American Journal of Diseases of Children*.

[25] Volk DM, Owen LG (1986). Cutaneous polyarteritis nodosa in a patient with ulcerative colitis. *Journal of Pediatric Gastroenterology and Nutrition*.

[26] Lightman HI, Valderrama E, Ilowite NT (1988). Cutaneous polyarteritis nodosa and thromboses of the superior and inferior venae cavae. *Journal of Rheumatology*.

[27] Praderio L, Corti C, Sposato E, Taccagni GL, Volpi A, Sabbadini MG (1989). Berger’s disease with polyarteritis nodosa. *British Journal of Rheumatology*.

[28] Moreland LW, Ball GV (1990). Cutaneous polyarteritis nodosa. *American Journal of Medicine*.

[29] Kondo N, Motoyoshi F, Ozawa T, Orii T (1991). A case report of a 9-year old boy with polyarteritis nodosa in which a combination therapy of corticosteroids and a photosensitive dye Platonin was effective. *Biotherapy*.

[30] Jorizzo JL, White WL, Wise CM, Zanolli MD, Sherertz EF (1991). Low-dose weekly methotrexate for unusual neutrophilic vascular reactions: cutaneous polyarteritis nodosa and Behcet’s disease. *Journal of the American Academy of Dermatology*.

[31] Draaisma JMT, Fiselier TJW, Mullaart RA (1992). Mononeuritis multiplex in a child with cutaneous polyarteritis. *Neuropediatrics*.

[32] Andresdottir MB, Sukhai R, Swaak AJG (1993). Polyarteritis nodosa in a young man, a ten-year delay in diagnosis. *Clinical Rheumatology*.

[33] Stone MS, Olson RR, Weismann DN, Giller RH, Goeken JA (1993). Cutaneous vasculitis in the newborn of a mother with cutaneous polyarteritis nodosa. *Journal of the American Academy of Dermatology*.

[34] Bakkaloglu A, Soylemezoglu O, Tinaztepe K, Saatci U (1994). A possible relationship between polyarteritis nodosa and hydatid disease. *European Journal of Pediatrics*.

[35] Drymalski WG, Hosen RS, Smook S (1994). Response to pooled gamma globulin therapy in a child with polyarteritis nodosa. *Archives of Pediatrics and Adolescent Medicine*.

[36] Gedalia A, Correa H, Kaiser M, Sorensen R (1995). Steroid sparing effect of intravenous gamma globulin in a child with necrotizing vasculitis. *American Journal of the Medical Sciences*.

[37] Fitzgerald DA, Verbov JL (1996). Cutaneous polyarteritis nodosa. *Archives of Disease in Childhood*.

[38] Verbov J (1980). Cutaneous polyarteritis nodosa in a young child. *Archives of Disease in Childhood*.

[39] Ginarte M, Pereiro M, Toribio J (1998). Cutaneous polyarteritis nodosa in a child. *Pediatric Dermatology*.

[40] Mocan H, Mocan MC, Peru H, Özoran Y (1998). Cutaneous polyarteritis nodosa in a child and a review of the literature. *Acta Paediatrica*.

[41] Albornoz MA, Benedetto AV, Korman M, McFall S, Tourtellotte CD, Myers AR (1998). Relapsing cutaneous polyarteritis nodosa associated with streptococcal infections. *International Journal of Dermatology*.

[42] Uziel Y, Silverman ED (1998). Intravenous immunoglobulin therapy in a child with cutaneous polyarteritis nodosa. *Clinical and Experimental Rheumatology*.

[43] Schrodt BJ, Callen JP (1999). Polyarteritis nodosa attributable to minocycline treatment for acne vulgaris. *Pediatrics*.

[44] Tonnelier J-M, Ansart S, Tilly-Gentric A, Pennec Y-L (2000). Juvenile relapsing periarteritis nodosa and streptococcal infection. *Joint Bone Spine*.

[45] Yetgin S, Ozen S, Yenicesu I, Çetin M, Bakkaloğlu A (2001). Myelodysplastic features in polyarteritis nodosa. *Pediatric Hematology and Oncology*.

[46] Falcini F, Lionetti P, Simonini G, Resti M, Cimaz R (2001). Severe abdominal involvement as the initial manifestation of cutaneous polyarteritis nodosa in a young girl. *Clinical and Experimental Rheumatology*.

[47] Bauzá A, España A, Idoate M (2002). Cutaneous polyarteritis nodosa. *British Journal of Dermatology*.

[48] Gargollo PC, Barnewolt CE, Diamond DA (2004). Calcified ureteral stricture in a child with polyarteritis nodosa. *Journal of Urology*.

[49] Hatemi G, Masatlioglu S, Gogus F, Ozdogan H (2004). Necrotizing vasculitis associated with familial Mediterranean fever. *American Journal of Medicine*.

[50] Faller G, Kala UK, Hale MJ (2005). Polyarteritis nodosa presenting with a leg mass. *Acta Paediatrica, International Journal of Paediatrics*.

[51] Klusmann A, Megahed M, Kruse R, Schneider M, Schmidt KG, Niehues T (2006). Painful rash and swelling of the limbs after recurrent infections in a teenager: polyarteritis nodosa. *Acta Paediatrica, International Journal of Paediatrics*.

[52] Tehrani R, Nash-Goelitz A, Adams E, Dahiya M, Eilers D (2007). Minocycline-induced cutaneous polyarteritis nodosa. *Journal of Clinical Rheumatology*.

[53] González-Fernández MA, García-Consuegra J (2007). Polyarteritis nodosa resistant to conventional treatment in a pediatric patient. *Annals of Pharmacotherapy*.

[54] Borrie P (1972). Cutaneous polyarteritis nodosa. *British Journal of Dermatology*.

[55] Magilavy DB, Petty RE, Cassidy JT, Sullivan DB (1977). A syndrome of childhood polyarteritis. *Journal of Pediatrics*.

[56] David J, Ansell BM, Woo P (1993). Polyarteritis nodosa associated with streptococcus. *Archives of Disease in Childhood*.

[57] Al Mazyad AS (1999). Polyarteritis nodosa in Arab children in Saudi Arabia. *Clinical Rheumatology*.

